# Modeling Hearing Loss Progression and Asymmetry in the Older Old: A National Population-Based Survey

**DOI:** 10.1093/geroni/igaa057.693

**Published:** 2020-12-16

**Authors:** Rahul Sharma, Anil Lalwani, Justin Golub

**Affiliations:** 1 Columbia University Irving Medical Center, New York, New York, United States; 2 NewYork-Presbyterian Hospital/Columbia University Irving Medical Center, New York City, New York, United States

## Abstract

The progression and asymmetry of age-related hearing loss has not been well characterized in those 80 years of age and older because public datasets mask upper extremes of age to protect anonymity. We aimed to model the progression and asymmetry of hearing loss in the older old using a representative, national database. This was a cross-sectional, multicentered US epidemiologic analysis using the National Health and Nutrition Examination Study (NHANES) 2005-2006, 2009-2010, and 2011-2012 cycles. Subjects included non-institutionalized, civilian adults 80 years and older (n=621). Federal security clearance was granted to access publicly-restricted age data. Outcome measures included pure-tone average air conduction thresholds and the 4-frequency pure tone average (PTA). 621 subjects were 80 years old or older (mean=84.2 years, range=80-104 years), representing 10,600,197 Americans. Hearing loss exhibited constant acceleration across the adult lifespan at a rate of 0.0052 dB/year2 (95% CI = 0.0049, 0.0055). Compounded over a lifetime, the velocity of hearing loss would increase five-fold, from 0.2 dB loss/year at age 20 to 1 dB loss/year at age 100. This model predicted mean PTA within 2 dB of accuracy for most ages between 20 and 100 years. There was no change in the asymmetry of hearing loss with increasing age over 80 years (linear regression coefficient of asymmetry over age=0.07 (95% CI=-0.01, 0.24). In conclusion, hearing loss steadily and predictably accelerates across the adult lifespan to at least age 100, becoming near-universal. These population-level statistics will guide treatment and policy recommendations for hearing health in the older old.

